# Supplementary material for: MetaRibo-Seq measures translation in microbiomes
Source: Nat Commun. 2020 Jun 29;11:3268. doi: 10.1038/s41467-020-17081-z (PMC7324362; doi:10.1038/s41467-020-17081-z)
Supplement: Supplementary file 10 — Supplementary Data 7 [file 41467_2020_17081_MOESM10_ESM.zip › File2/Confidence_VeryHigh_Taxonomy/30886_out.krona.html]

Javascript must be enabled to view this page.

members
magnitude
magnitudeUnassigned
count
unassigned
taxon
rank

30886\_out

16


SRS014979\_contig\_number\_contig-100\_26036.70744SRS015578\_contig\_number\_contig-100\_9537.9537SRS065397\_contig\_number\_contig-100\_11514.11515
3

superkingdom
13
2

phylum
1
1224

class
1
28211

order
204441
1

1
41295
family

genus
191
1

species

SRS148874\_contig\_number\_16072
1896972
1

12
1239
phylum

186801
12
class

186802
12
4

SRS011405\_contig\_number\_contig-100\_3353.41456SRS013951\_contig\_number\_10681SRS018984\_contig\_number\_contig-80\_1484.239352SRS053398\_contig\_number\_contig-100\_7102.111379
order

family
6
541000

genus
6
946234

1193534
5
species

SRS015890\_contig\_number\_10994SRS098571\_contig\_number\_32181SRS098717\_contig\_number\_18575SRS103987\_contig\_number\_33580SRS140492\_contig\_number\_contig-100\_443.203189

292800
1
species

SRS049402\_contig\_number\_17443

31979
1
family

genus
1
1485

649724
1
species

SRS149244\_contig\_number\_12788

216572
1
family

1
459786
genus

species

SRS017521\_contig\_number\_29518
2109687
1
